# Immunotoxicity Study of Cucurbit[n]urils (n = 6, 7, 8) and Modeling of Interaction with Some Monocyte Receptors by a Molecular Docking Method

**DOI:** 10.3390/molecules30102249

**Published:** 2025-05-21

**Authors:** Saule B. Zhautikova, Nursipat N. Abdykhanova, Dmitry A. Fedorishin, Yelena G. Shapovalova, Andrei I. Khlebnikov, Abdigali A. Bakibaev, Irina A. Kurzina, Saule K. Kabieva, Nazerke Boranbay, Gaziza M. Zhumanazarova

**Affiliations:** 1 Karaganda Medical University, Karaganda 100900, Kazakhstan; zhautikova@qmu.kz; 2 Karaganda Industrial University, Temirtau 101400, Kazakhstan; kabieva.s@mail.ru (S.K.K.); n.boranbay@tttu.edu.kz (N.B.); gaziza.zhumanazarova@mail.ru (G.M.Z.); 3 Department of Natural Compounds, Chemical Faculty, Pharmaceutical and Medical Chemistry, National Research Tomsk State University, Tomsk 634050, Russia; strix187@yandex.ru (D.A.F.); egshapovalova@yandex.ru (Y.G.S.); aikhl@chem.org.ru (A.I.K.); kurzina99@mail.ru (I.A.K.); 4 National Research Tomsk Polytechnic University, Tomsk 634050, Russia

**Keywords:** cucurbit[6]uril, cucurbit[7]uril, cucurbit[8]uril, monocytes, cytotoxicity, immunotoxicity, Toll-like receptors, molecular docking

## Abstract

In this study, cucurbit[n]urils (n = 6, 7, 8) were carefully evaluated for their cytotoxicity and immunotoxicity to human peripheral blood monocytes. The cytotoxicity was studied by evaluating the survival of monocytes, while the immunotoxicity level was assessed by analyzing the inflammatory mediators secreted by them using an enzyme-linked immunosorbent assay. It was found that cucurbit[n]urils (n = 6, 7, 8) in the used concentration (10^−5^ M) do not cause a negative effect on cell viability, which is maintained at a level above 50%. At the same time, cucurbit[n]urils (n = 6, 7, 8) do not cause pro-inflammatory activation of monocytic macrophages. The absence of stimulation of pro-inflammatory cytokine expression demonstrates the promising biocompatibility of the studied compounds, which is crucial for their successful clinical use. The obtained results of molecular modeling show the possibility of formation of **CB[6]**, **CB[7]**, and **CB[8]** associates with various Toll-like receptors, which also confirms good prospects for the development of new ways of medical application of cucurbit[n]urils.

## 1. Introduction

In the modern era, personalized medicine is becoming increasingly relevant, for which there is an urgent need to develop new pharmacological agents and biomaterials for medical use. Targeted drug delivery is of paramount importance in modern medicine due to the possibility of increasing therapeutic efficacy while minimizing side effects. Traditional drug delivery methods often lack sufficient specificity, resulting in the systemic distribution of drugs, which can result in suboptimal drug concentrations at the disease focus and significant off-target toxicity leading to serious side effects. Targeted delivery systems address this limitation by directing therapeutic agents specifically to diseased tissues or cells, thereby improving the pharmacokinetic and pharmacodynamic profiles of drugs [1].

This approach is particularly important in the treatment of complex diseases such as cancer, where traditional chemotherapeutic agents often cause serious side effects due to their effects on healthy, rapidly dividing cells. Targeted systems, such as antibody–drug conjugates or nanoparticles, allow selective drug accumulation at the tumor site through mechanisms such as receptor-mediated endocytosis or enhanced permeability and retention (EPR) effects [2].

In addition, targeted drug delivery facilitates the use of advanced therapies, including gene editing tools, RNA-based drugs, and immunotherapy, which require precise localization for therapeutic effects. By improving treatment precision and reducing systemic toxicity, targeted drug delivery meets the broader goals of personalized medicine, thereby meeting the growing demand for safer and more effective therapeutic strategies in modern healthcare [1].

Recently, researchers have focused their efforts on the creation of new supramolecular systems based on macrocyclic compounds. One of the promising classes of such compounds is the cucurbit[n]urils (**CB[n]s**). They have an internal hydrophobic cavity capable of holding molecules or ions due to non-covalent interactions such as hydrogen bonds, hydrophobic effects, and van der Waals forces [3]. The utilization of the hydrophobic **CB[n]** cavity for the assembly of biologically active functional molecules has attracted much research attention. This interest extends beyond its role as a targeted drug delivery system to applications in disease diagnosis and various other fields [4,5]. Currently, antitumor, antibacterial, and anticholinergic drugs; antioxidants; neurotransmitters; cholinesterase reactivators; and other compounds are encapsulated in the **CB[n]** cavity [3,4,5].

Targeting of the cucurbit[n]uril–drug system can be achieved by appropriate modification of cucurbit[n]uril with functional groups that are recognized by specific receptors located on the cell surface [6,7]. Thus, **CB[n]s** have become unique and intriguing molecules for the development of targeted delivery systems with multifaceted applications in various fields.

In order to develop targeted drug delivery systems, the search for biological targets for point targeting is essential. One of the methods for searching for targets and modeling their interaction with ligands is in silico molecular modeling, such as molecular docking, which is widely used in drug discovery and development due to its ability to efficiently screen large libraries of compounds and provide insight into the molecular basis of ligand–target interaction. Such a method can refine the chemical structure of leading compounds by identifying modifications that increase binding affinity and specificity [8].

We have previously found that the application of cucurbit[n]urils (n = 6, 7 8) to porous medical-grade materials increases monocyte survival and decreases the level of pro-inflammatory cytokines secreted by them [9]. To elucidate the most likely mode of interaction of cucurbit[n]urils (**CB[6]**, **CB[7]**, and **CB[8]**) with monocyte receptors, this study involved the molecular docking of these compounds to TLR1 [10], TLR3 [11], TLR4 [12], TLR5 [13], and TLR8 [14] receptors. Accordingly, the main aim of this study is to evaluate the effects of **CB[6]**, **CB[7]**, and **CB[8]** on monocyte survival and their secretion of pro-inflammatory cytokines, and to molecularly model the interaction of these cucurbit[n]urils with some receptors of innate immunity.

## 2. Results and Discussion

### 2.1. Cytotoxicity Study of the Samples

In the next step of the study, a monocyte–macrophage test system was used to evaluate immunotoxicity, and a concentration of **CB[6]**, **CB[7]**, **or CB[8]** of 1 × 10^−5^ M was used.

**Figure 1:**
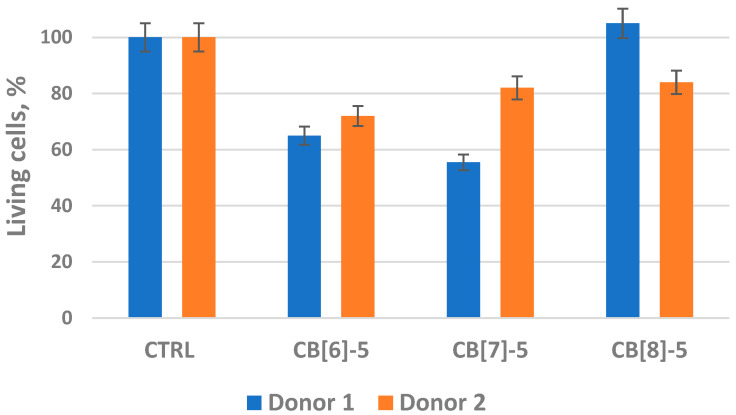
Viability assessment of primary human macrophages after 6 days of culturing in the presence of cucurbit[n]urils. Error bars represented the standard error of the mean and were calculated based on three biological repeats for a 0.05 level of significance. **CTRL**—control; **CB[6]-5**, **CB[7]-5**, **CB[8]-5**—cucurbit[n]urils at the respective concentrations indicated in Table 1.

As can be seen from the data of Figure 1, compounds **CB[6]**, **CB[7]**, and **CB[8]** at the concentration used had a toxic effect on primary human macrophages (*p* < 0.05). However, it should be noted that the viability of macrophages is above 50% relative to the control (cells on substrate without samples). The **CB[8]-5** sample showed minimal cytotoxicity, in the case of **Donor 2** even increasing cell survival. The differences in cell survival between **Donor 1** and **Donor 2** may, in the case of the samples **CB[7]-5** and **CB[8]-5,** be due to the different reactivity of macrophages.

**Table 1:** Samples submitted for research.

№	Sample	Concentration, mol/L	Designation
**1**	**CB[6]**	1 × 10^−5^	**CB[6]-5**
**2**	**CB[7]**	1 × 10^−5^	**CB[7]-5**
**3**	**CB[8]**	1 × 10^−5^	**CB[8]-5**

Monocytes constitute 10% of leukocytes in human blood; in addition to playing an important role in development, homeostasis, and inflammation, they are also responsible for the removal of apoptotic and necrotic cells as well as bacterial pathogens [15]. Monocytes originate from hematopoietic stem cell monoblasts, which first differentiate into promonocytes and then into mature monocytes. It was previously suggested that circulating blood monocytes are the exclusive precursors of tissue macrophages [16]. Bone marrow-derived monocytes differentiate into macrophages in the gut and dermis during acute infection and inflammation [17]. Although macrophages in tissues share many common features, they are nevertheless extremely heterogeneous in terms of functional and surface marker expression [18]. Macrophages and their activation states are characterized by plasticity and flexibility [19,20]. Depending on their environment, macrophages have a wide range of functions, especially in modulating the innate immune response by secreting several factors. Signals received from the microenvironment, such as microbes, injured tissues, or activated lymphocytes, activate functional reprogramming of macrophages that lead to a spectrum of different functional phenotypes (e.g., the pro-inflammatory or anti-inflammatory nature of macrophages in immune responses) [21,22]. In particular, IFNγ or IFNγ alone, together with microbial stimuli (e.g., lipopolysaccharides (LPSs)) or cytokines (e.g., tumor necrosis factors (TNFs) and granulocyte colony-stimulating factor (GM-CSF)), induce classically activated pro-inflammatory M1 macrophages. Such classical activation is inhibited by IL-4 and IL-13, which in turn induce an alternative M2 form of macrophage activation [23].

The M1 phenotype is characterized by high levels of pro-inflammatory cytokines and high concentrations of nitrogen and oxygen intermediates. The pro-inflammatory profile of M1 macrophages is characterized by high levels of IL-12, IL-23, and inflammatory cytokines such as IL-1β, TNF, and IL-6, and low production of immunosuppressive cytokines such as IL-10. M1 macrophages classically participate in polarized Th1 responses, and show strong microbicidal and tumor cell activity, thus acting as effectors of body resistance against tumors and intracellular parasites [24]. The classical M1 phenotype of activated macrophages is well described in the literature; however, data regarding the M2 phenotype are rather contradictory. M2 macrophages are known to be characterized by low levels of expression of the pro-inflammatory cytokines IL-12 and IL-23, expressing high levels of IL-1 and showing a variable ability to secrete inflammatory cytokines. In contrast to pro-inflammatory M1 macrophages, M2 phenotype cells retain poor antigen-presenting potential and possess immunoregulatory functions such as suppression of Th1 adaptive immunity, active debris removal, stimulation of wound healing, angiogenesis, and tissue remodeling. Compared to M1 macrophages, alternatively activated cells express and produce lower amounts of IL-1β and high amounts of IL-1ra receptor [25].

### 2.2. Analysis of Pro-Inflammatory Cytokine Secretion by Monocytes in Response to Sample Effects

The next stage of the study was to investigate the pro-inflammatory activation of macrophages in response to the action of cucurbit[n]urils. As follows from the above, the sign of macrophage activation is the release of cytokines. Therefore, the choice of key macrophage cytokines such as TNF-α, IL-6, IL-10, IL-1β, and IL-8 as the main parameters to evaluate the immunomodulatory properties is consistent with the experimental model used in this study. Since the biological activity of cytokines is determined more by their concentration in the extracellular environment rather than by their expression level at the gene level, an enzyme-linked immunosorbent assay (ELISA) was chosen as the main analytical method to study the effect of the studied cucurbit[n]urils (n = 6, 7, 8) on cytokine production.

Tumor necrosis factor-alpha (TNF-α) is a multifunctional pro-inflammatory cytokine and extracellular protein predominantly synthesized by monocytes and macrophages. TNF-α plays a key role as a major mediator of inflammation and an important regulator of the immune response to infections and tumor development. In addition to its involvement in inflammatory processes, TNF-α influences lipid metabolism, blood coagulation, insulin resistance, and endothelial function. In addition, TNF-α stimulates the production of other cytokines such as IL-1, IL-6, IL-8, and interferon-gamma, and activates leukocytes, playing an important role in the body’s defense against intracellular pathogens and viruses [26].

Interleukin-6 (IL-6) is a key multifunctional cytokine. It plays an important role in the differentiation of activated B-lymphocytes into plasma cells that secrete immunoglobulins and regulates the acute phase of the inflammatory response. Elevated levels of IL-6 are frequently observed in various inflammatory conditions and correlate with laboratory markers of systemic inflammation [25,27].

Interleukin-10 (IL-10), which belongs to class 2 cytokines, is characterized by potent anti-inflammatory properties. IL-10 suppresses the production of pro-inflammatory cytokines such as IFN-γ, TNF-α, IL-1β, and IL-6 in various cell types. Moreover, it inhibits dendritic cell maturation by partially suppressing IL-12 expression. In addition to its anti-inflammatory functions, IL-10 also exerts immunostimulatory effects. It is able to stimulate IFN-γ production by CD8+ T cells activated using anti-CD3/anti-CD28 or cytokine cocktails. In addition, IL-10 is a potent growth and differentiation factor for various immune cells including B cells, mast cells, and thymocytes [28].

Interleukin-1 beta (IL-1β) is a cytokine that plays a central role in the regulation of inflammatory responses. Its functions encompass immunomodulatory, hematopoietic, inflammatory, and intersystem processes. IL-1β is important for triggering early immune responses, especially by activating T helper cells and engaging specific T-lymphocytes. In addition, IL-1β promotes the differentiation of B-lymphocytes into plasma cells, accelerating antibody production. This cytokine is one of the first participants in the body’s defense responses to pathogens, coordinating and regulating inflammatory and immune responses. IL-1β activates neutrophils and T- and B-lymphocytes, and stimulates the synthesis of acute phase proteins. It also enhances phagocytosis, hematopoiesis, and vascular permeability and has cytotoxic and bactericidal properties. In addition, IL-1β increases neutrophil mobility and cell activity in inflammatory foci, in addition to enhancing the effect of other cytokines, playing a significant role in the development of the inflammatory cascade [25].

Interleukin-8 (IL-8), also known as CXCL8, is a chemoattractant cytokine belonging to the CXC family of chemokines. It is secreted by a variety of cells, including monocytes, macrophages, neutrophils, fibroblasts, keratinocytes, and endothelial cells, in response to stimuli such as bacterial products, viral infections, pro-inflammatory cytokines (e.g., interleukin-1β or tumor necrosis factor-alpha), and hypoxia. IL-8 is a potent chemoattractant that attracts neutrophils to the focus of inflammation. This allows rapid mobilization of immune cells to defend the body against infections and tissue damage [29]. IL-8 activates neutrophils by enhancing their adhesion to the endothelium, migration through the vascular wall and phagocytic activity. It also stimulates the release from neutrophils of oxygen radicals and proteolytic enzymes necessary for pathogen destruction. IL-8 also stimulates the proliferation and migration of endothelial cells, which promotes angiogenesis (formation of new blood vessels). This function plays an important role in tissue healing and tumor growth. IL-8 enhances inflammation by regulating the activity of other cytokines and attracting additional immune cells, including monocytes and lymphocytes, to the inflammatory focus, and also participates in the body’s defense against bacterial and viral infections by regulating the migration and activation of immune cells [30].

**Figure 2:**
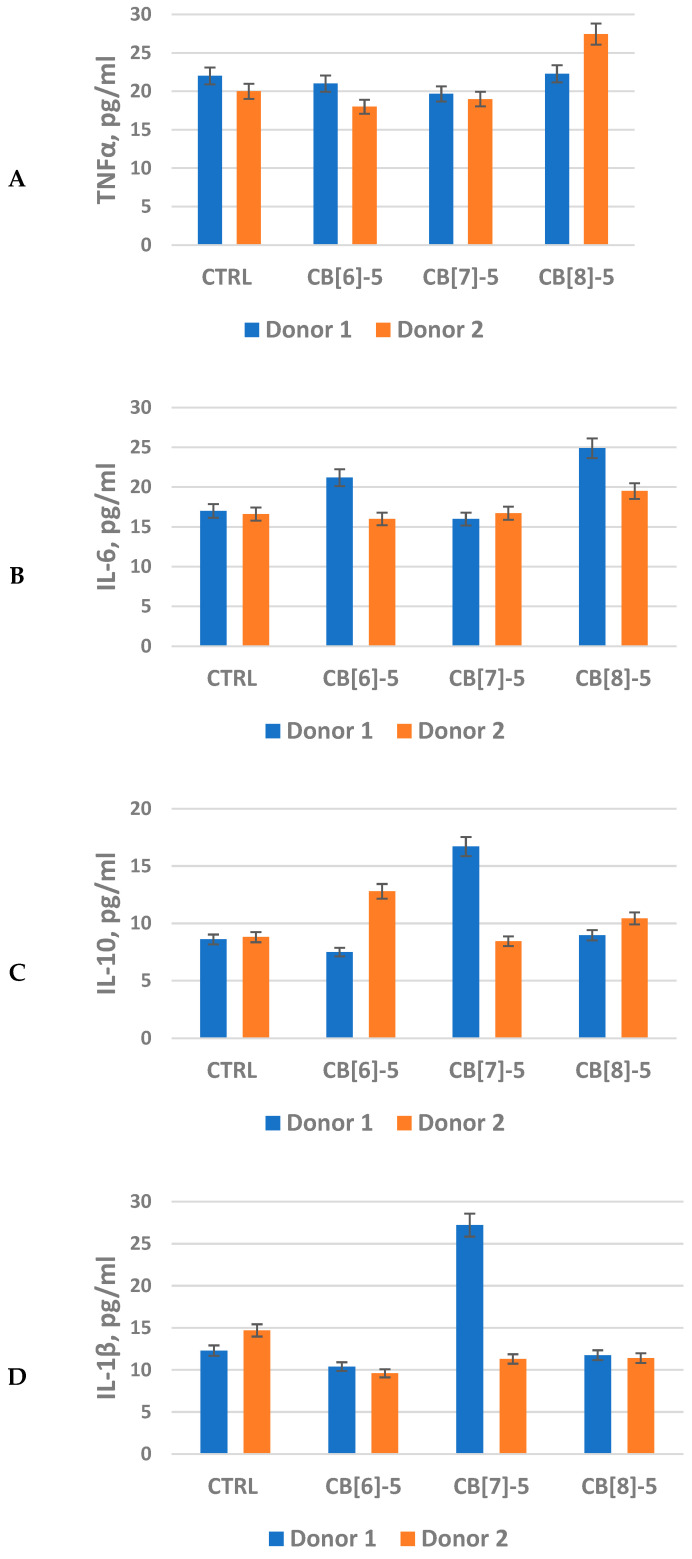

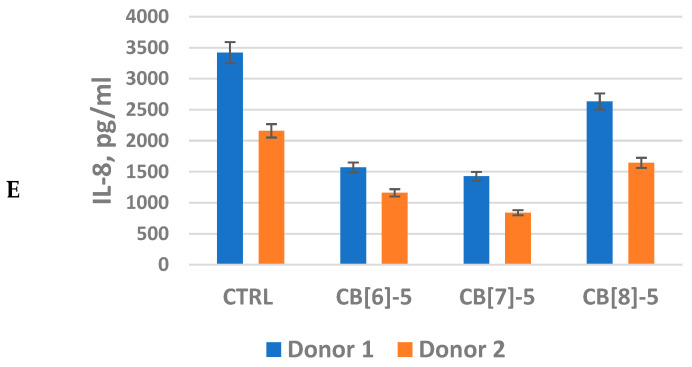
Effect of cucurbit[n]uril on the production of pro-inflammatory cytokines by primary human monocytic macrophages. Error bars represent the standard error of the mean and were calculated based on three biological repeats for a 0.05 level of significance. (**A**)—TNFα; (**B**)—IL-6; (**C**)—IL-10; (**D**)—IL-1β; (**E**)—IL-8; **CTRL**—control; **CB[6]-5**, **CB[7]-5**, **CB[8]-5**—cucurbit[n]urils at the respective concentration indicated in Table 1.

The study of the effects of **CB[6]**, **CB[7]**, and **CB[8]** on TNFα expression showed that during the whole period of cultivation, the concentration of the analyzed cytokine is at the level of control and does not exceed 25 pg/mL. It can be concluded that cucurbit[6]uril samples at the selected concentrations do not cause an increase in the inflammatory cytokine TNFα (Figure 2A).

The study of IL-6 secretion by monocytes showed that the tested samples do not cause a significant increase in IL-6 production on the sixth day of cultivation by macrophages of all donors compared to the control (Figure 2B).

Examination of IL-10 secretion showed that the cytokine concentration when donor cells 1 and 2 were cultured in the presence of **CB[6]**, **CB[7]**, or **CB[8]** was determined at the control level (Figure 2C).

Evaluation of IL-1β secretion showed that the cytokine concentration when cells were cultured in the presence of **CB[6]**, **CB[7]**, and **CB[8]** was determined at the control level (Figure 2D).

Analysis of IL-8 secretion showed that the cytokine was expressed both when cells were seeded on plastic and in the presence of the analyzed samples (Figure 2E) and reached 3420 pg/mL in the case of seeding Donor 1 cells on plastic during cultivation. At the same time, the concentration of the analyzed cytokine in the presence of **CB[6]**, **CB[7]**, and **CB[8]** is lower than in the control. The discrepancies in the IL-8 concentrations after incubation with **CB[6]**, **CB[7]**, and **CB[8]** may be due to the different initial level of donor immune reactivity.

The lack of significant differences in cytokine concentrations after incubation with **CB[6]**, **CB[7]**, and **CB[8]** indicates that they do not induce inflammation or immune response at the selected concentration within the experimental conditions. It can be concluded that individual differences in immune reactivity should be taken into account when designing carriers for targeted drug delivery. This parameter may influence cytokine secretion levels, emphasizing the need to consider patient-specific factors, especially when evaluating pharmacological parameters and immune responses.

As follows from the above, cucurbit[n]urils are stable molecules with low immune reactivity, which is one of the factors that make them suitable for use as a carrier for targeted drug delivery. In the next phase of the study, the putative mechanism of mononuclear cell activation in silico was investigated.

As follows from the literature, the process of mononuclear cell activation begins with the interaction of ligand with Toll-like receptors located on their membrane [10,31]. We suggested that the decrease in the level of pro-inflammatory cytokine expression is associated with the interaction of cucurbit[n]uril molecules with Toll-like receptors, triggering a signaling cascade that ultimately leads to a change in the level of pro-inflammatory cytokine secretion.

### 2.3. Analysis of the Affinity of CB[6], CB[7], and CB[8] with Some Toll-Like Receptors of Monocytes (TLR1, TLR3, TLR4, TLR5, and TLR8) by Molecular Docking Method

Toll-like receptors (TLRs) belong to a class of pattern recognition receptors (PRRs) that play a key role in the innate immune system by recognizing conserved molecular patterns associated with microbial pathogens, known as pathogen-associated molecular patterns (PAMPs), and damaging molecular patterns (DAMPs) from host cells. Toll-like receptors are transmembrane proteins located on the surface or in the endosomes of various immune cells such as macrophages, dendritic cells, and neutrophils, as well as non-immune cells [31]. TLRs recognize specific pathogens such as bacterial lipopolysaccharides, virus nucleic acids, etc. After ligand binding, TLRs initiate signaling cascades through adaptor proteins such as MyD88 (myeloid differentiation primary response 88) or TRIF (TIR-domain-containing adaptor-inducing interferon-β). These pathways lead to the activation of transcription factors such as NF-κB and IRFs (interferon regulatory factors), promoting the production of pro-inflammatory cytokines, type I interferons, and antimicrobial peptides. TLR activation enhances antigen presentation and expression of co-stimulatory molecules on dendritic cells, promoting activation of T cells and B cells. This bridge between innate and adaptive immunity is essential for generating robust and specific immune responses. Toll-like receptors are central to innate immunity, detecting bacteria and cellular damage, thereby organizing a coordinated immune response. Their diverse functions make them crucial targets for therapeutic intervention in a wide range of diseases [32].

TLR1 forms heterodimers with TLR2 to recognize a wide range of microbial components, particularly triacylated lipoproteins from bacterial and mycobacterial cell walls. This receptor recognizes bacterial lipoproteins and promotes an inflammatory response. It triggers the MyD88-dependent signaling pathway, leading to the activation of NF-κB and the production of pro-inflammatory cytokines such as TNF-α and IL-6, and plays a key role in the recognition of Gram-positive bacteria, mycobacteria, and some fungi [10,30].

TLR3 detects viral RNA, triggering an antiviral immune response. It activates the TRIF-dependent signaling pathway (different from the MyD88 pathway), leading to the production of type I interferons (e.g., IFN-α, IFN-β) and pro-inflammatory cytokines. Also, this receptor plays a key role in combating RNA virus infections (e.g., reoviruses) and is important for enhancing dendritic cell maturation and activation of CD8+ T cells [11].

TLR4 recognizes bacterial lipopolysaccharides and initiates a potent pro-inflammatory response. This receptor activates MyD88-dependent and TRIF-dependent pathways, which leads to the production of inflammatory cytokines (e.g., TNF-α, IL-1β) and interferons (e.g., IFN-β), plays a critical role in the immune response to sepsis and other systemic bacterial infections, and promotes tissue repair and homeostasis, but may also mediate chronic inflammation in diseases such as atherosclerosis and rheumatoid arthritis [12].

TLR5 detects flagellar bacteria and contributes to host defense by inducing inflammatory cytokines and antimicrobial peptides and signaling through MyD88-dependent pathway to activate NF-κB and MAPK, leading to the production of cytokines such as IL-6 and TNF-α. Also, this receptor plays an important role in gut immunity by maintaining homeostasis of the gut microbiota and responding to invasive flagellar pathogens (e.g., *Salmonella* and *Escherichia coli*) [31].

TLR8 recognizes viral single-stranded RNAs, especially during endosomal processing of pathogens and signals through the MyD88-dependent pathway by activating NF-κB and IRF7, leading to the production of pro-inflammatory cytokines (e.g., TNF-α, IL-6) and antiviral interferons [31]. It plays an important role in the immune response to RNA viruses (e.g., influenza, HIV). This receptor also promotes the activation of monocytes, macrophages, and dendritic cells [32].

To evaluate the interaction of cucurbit[6]uril (**CB[6]**), cucurbit[7]uril (**CB[7]**), and cucurbit[8]uril (**CB[8]**) with various proteins in silico, we performed molecular docking of **CB[6]**, **CB[7]**, and **CB[8]** compounds into protein structures with the following PDB codes: 6NIH (TLR1 receptor), 1ZIW (TLR3), 5NAO (TLR4), 8AR2 (TLR5), and 3W3G (TLR8). The aim of the computational experiments was to determine the likely position of cucurbit[n]urils when binding to a particular biom target, as well as the strength of this binding using the DS (Docking Score in the MolDock force field) evaluation function.

**Figure 3:**
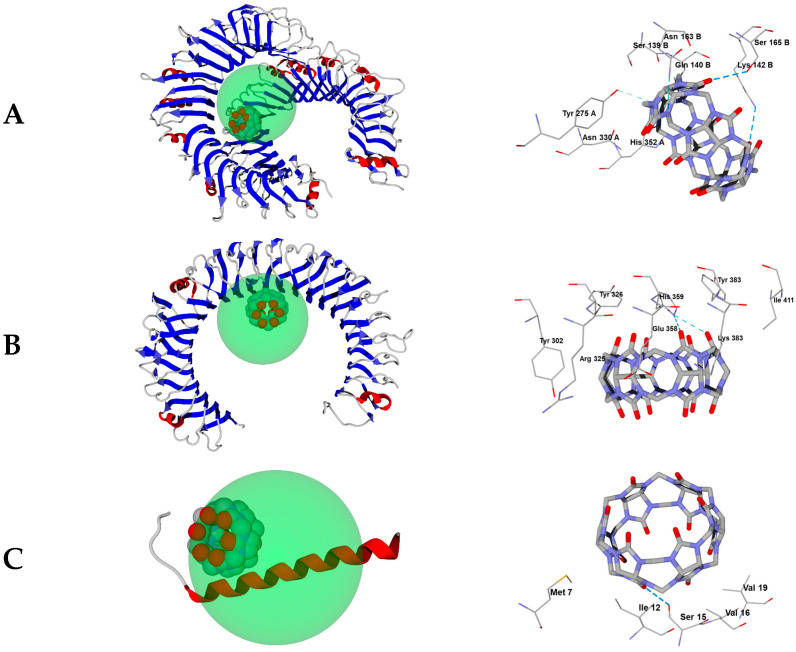

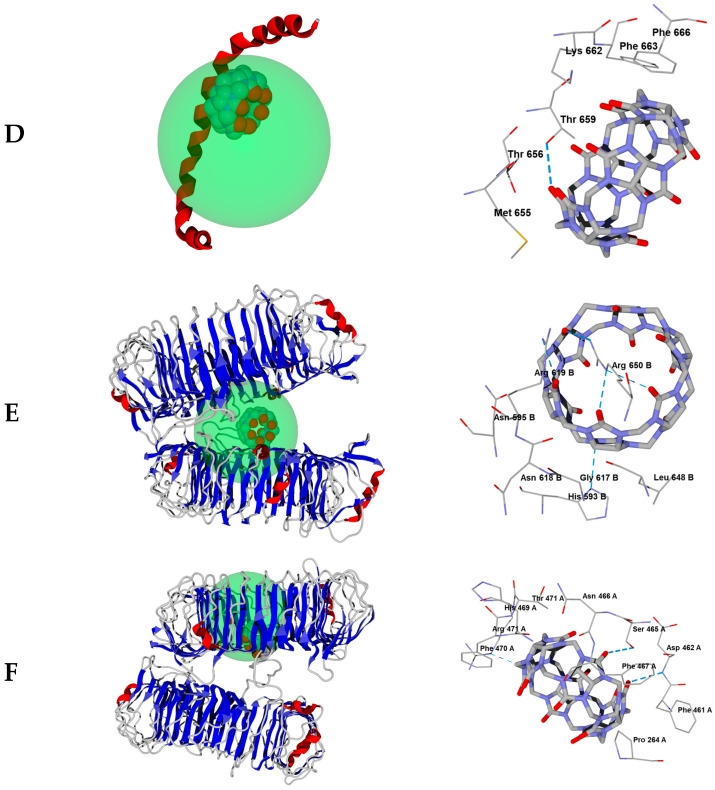
Best positions of the **CB[6]** molecule obtained by docking into PDB structures 6NIH (**A**), 1ZIW (**B**), 5NAO (**C**), 8AR2 (**D**), 3W3G with a search region between two protomers (**E**), and 3W3G with a search region within one of the protomers (**F**). On the left, docking positions are shown together with spherical search regions of 15 Å radius and with the protein displayed as ribbon diagrams. The search regions are shown as green spheres. On the right, docking positions are shown surrounded by the nearest amino acid residues (within 3 Å) with hydrogen bonding displayed as blue dashed lines.

**Figure 4:**
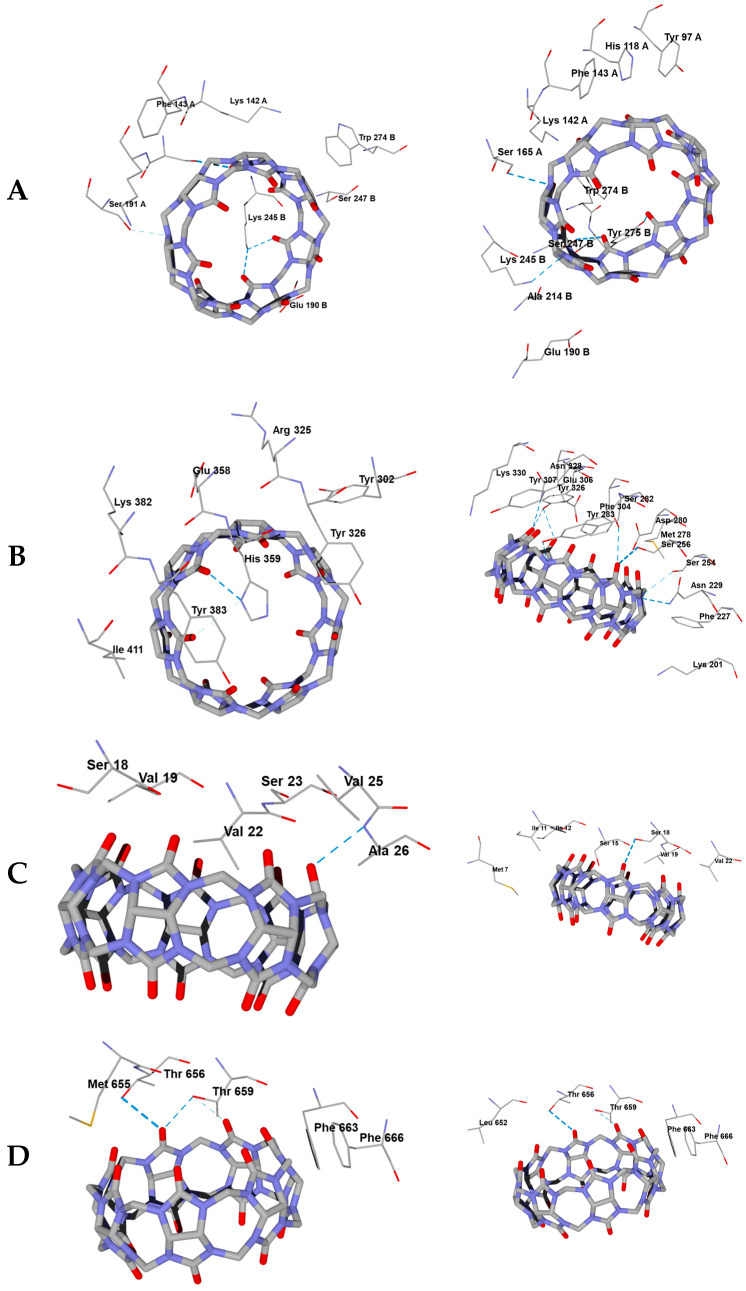

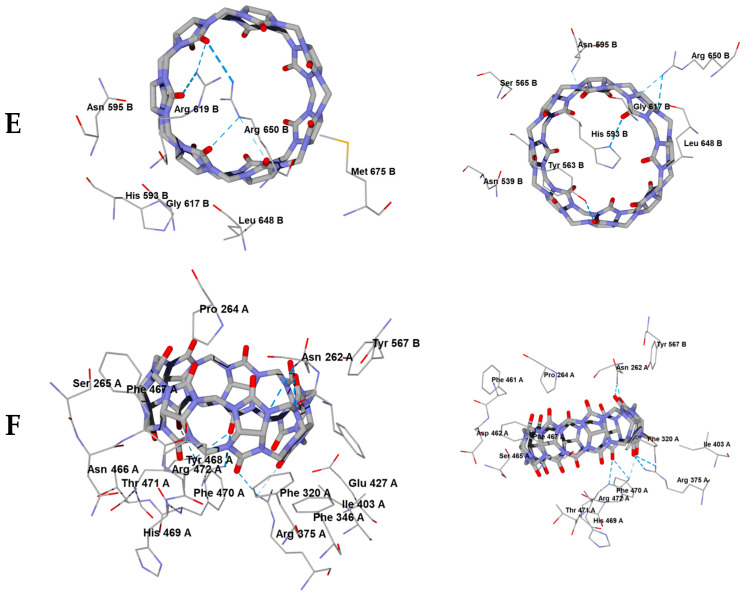
Best positions of **CB[7]** (**left**) and **CB[8]** (**right**) molecules obtained by docking into PDB structures 6NIH (**A**), 1ZIW (**B**), 5NAO (**C**), 8AR2 (**D**), 3W3G with a search region between two protomers (**E**), and 3W3G with a search region within one of the protomers (**F**). Docking positions are shown surrounded by the closest amino acid residues (within 3 Å) with hydrogen bonds shown as blue dashed lines.

**Figure 5:**
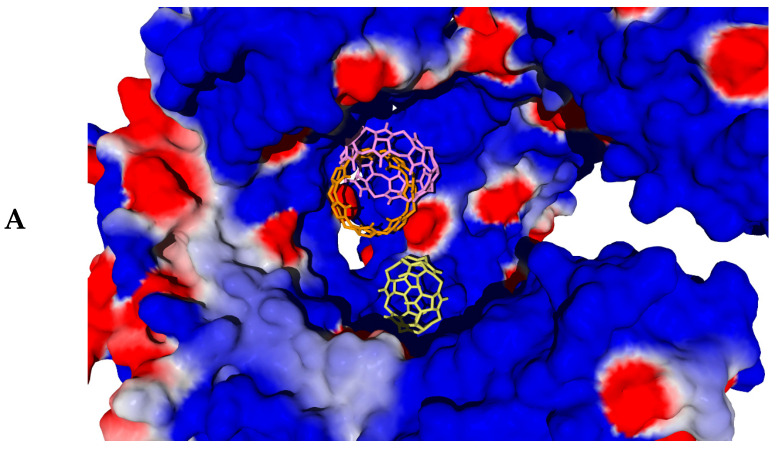

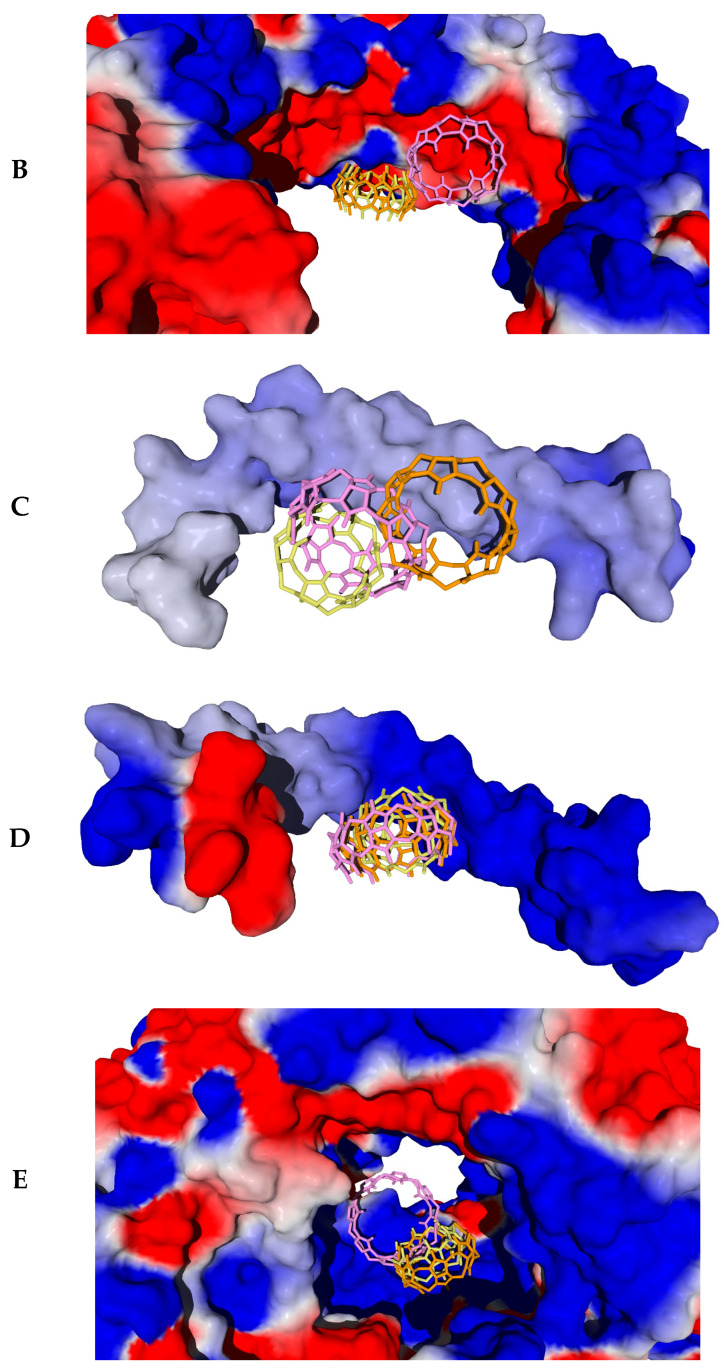

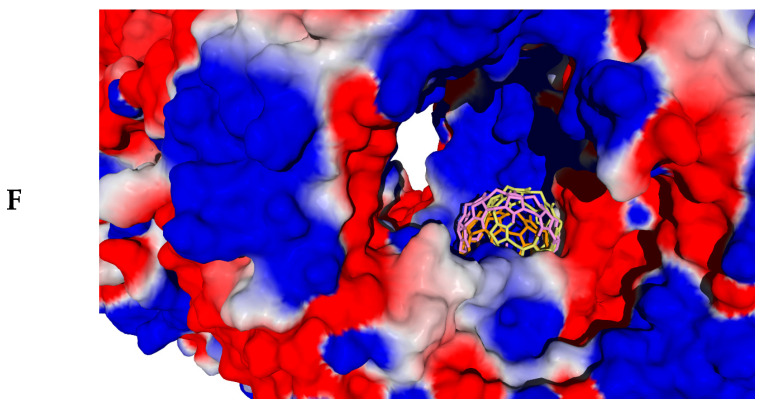
Aligned docking positions of **CB[6]** (yellow) **CB[7]** (orange), and **CB[8]** (pink) molecules obtained by docking into PDB structures 6NIH (**A**), 1ZIW (**B**), 5NAO (**C**), 8AR2 (**D**), 3W3G with a search region between two protomers (**E**), and 3W3G with a search region within one of the protomers (**F**). Docking positions are shown against the protein surface. Red surface regions correspond to excess negative charge; blue regions correspond to excess positive charge.

The molecular docking computational projects, each relating to a specific protein structure together with a fixed search area (see Section 3.3), resulted in the best docking positions of ligands **CB[6]**, **CB[7]**, and **CB[8]**, which are shown in Figure 3, Figure 4 and Figure 5. The DS values and the hydrogen bonds formed between the ligands and the protein are summarized in Table 2.

The molecular containers **CB[6]**, **CB[7]**, and **CB[8]** form strong hydrogen bonds (H-bonds) with some amino acid residues when binding to the studied proteins, mainly with the participation of carbonyl oxygen atoms on the portals of the container. Some residues within the 6NIH, 1ZIW, and 3W3G proteins also form weak hydrogen bonds with nitrogen atoms of glycoluryl moieties (Table 2). In the case of **CB[6]**, the exception is the histidine residue His593B of the 3W3G protein, which interacts with the glycoluryl nitrogen atom through a rather strong H-bond (Figure 3E). The **CB[7]** molecule involving a nitrogen atom forms a very strong hydrogen bond with the Asn262A residue of protein 3W3G. As for the **CB[8]** compound, most of the H-bonds with nitrogen atoms of this ligand are strong, except for those formed with residues Lys245B of protein 6NIH and Ser254 of receptor 1ZIW. However, as noted above, hydrogen bonding of all three molecular containers studied to Toll-like receptors is mainly at the expense of carbonyl oxygen atoms on the portals, which is generally characteristic of the interaction of cucurbit[n]urils with polar sites.

In the case of the dimeric 6NIH protein structure, where the spherical search region is in the vicinity of both protomers, residues of both protomer A and protomer B participate in hydrogen bonding to ligands (Table 2, Figure 3A, Figure 4A and Figure 5A). At the same time, in one of the computational projects related to biotarget 3W3G, despite the localization of the search region between the two protomers with a toroidal tertiary structure (Figure 3E, Figure 4E and Figure 5E), only residues of protein chain B are involved in hydrogen bonding with ligands (Table 2). It should be noted that the retention of **CB[6]**, **CB[7]**, and **CB[8]** molecules by the investigated proteins occurs not only with the participation of H-bonds, but also due to van der Waals and electrostatic interactions of ligands with the nearest amino acid residues shown in Figure 3A–F, Figure 4A–F and Figure 5A–F. Thus, despite the strong hydrogen bonding of **CB[6]** to the TLR8 protein (structure 3W3G) when the ligand is placed in the region between protomers (Figure 3), the alternative form of interaction with TLR8 (Figure 3F) is characterized by a more negative DS value (Table 2), although fewer H-bonds are formed in this case. For all biotargets studied, there is a tendency for the absolute DS value to increase with an increasing number of glycoluryl links in the ligand (Table 2), consistent with a greater capacity for van der Waals interactions and for hydrogen bond formation when moving from **CB[6]** to **CB[7]** and on to **CB[8]**.

The docking positions of cucurbit[n]urils (n = 6, 7, 8) with different macrocycle sizes are approximately in the same region of space when binding to each particular protein (see Figure 5A–F with ligand positions aligned against the protein surface). Some deviations in the positions of **CB[6]** (6NIH—Figure 3A), **CB[7]** (5NAO—Figure 4C), and **CB[8]** (1ZIW—Figure 4B, 3W3G—Figure 4E) are not too significant and are within the spherical search region of 15 Å radius.

Overall, the molecular docking results indicate that cucurbit[n]urils (n = 6, 7, 8) can bind to the investigated proteins. At least, the estimated DS function for the docking positions found takes significantly negative values, in most cases exceeding 200 units in absolute value. The exception is the small protein TLR4 (5NAO structure), for which the value of DS = −189 was obtained by docking **CB[6]**, but even this value indicates a noticeable affinity of the **CB[6]** molecule to this protein.

A limitation of the approach used is that the conformational mobility of protein macromolecules (rigid biotarget approximation) was not taken into account in the computational experiment. On the other hand, taking into account the conformational adjustment of the protein to the ligand would lead to estimates that are even more in favor of the efficient interaction of cucurbit[n]urils with the studied Toll-like receptors.

As suggested by the in silico computational model, cucurbit[n]urils have an affinity for Toll-like receptors (TLRs). TLRs are involved in triggering inflammatory processes and blocking them can reduce inflammation. It can be assumed that cucurbit[n]uril molecules can bind to them in a nonspecific manner, competitively occupying the active center and inhibiting the initiation of the signaling cascade responsible for the activation of pro-inflammatory cytokine production, which is confirmed by cytotoxicity and ELISA assays.

## 3. Materials and Methods

Synthesis and determination of the composition of the studied cucurbit[n]urils by physicochemical methods of analysis were carried out according to the methodology developed by us [9].

At this stage of the study, the immunotoxicity of **CB[6]**, **CB[7]**, and **CB[8]** was investigated by assessing the viability of human mononuclear cells by reaction with AlamarBlue and analyzing the inflammatory mediators secreted by them using an enzyme-linked immunosorbent assay. The composition of the subject samples and their concentrations are summarized in Table 1. Concentrations were selected according to literature data [33,34,35], and solutions and suspensions were prepared using 70% ethyl alcohol, which, according to preliminary experiments, did not significantly affect cell viability at the concentration used. As a control, cells cultured on plastic without samples were studied.

### 3.1. Study of Cytotoxicity of Samples

Human monocytes isolated from the whole blood of a healthy male donor were used as test objects. Monocytes were isolated and cultured according to the method described in Kzhyshkowska J. et al. [36]. Cell culture condition and confluency were monitored using an Olympus CKX53 microscope (Olympus Corporation, Tokyo, Japan).

To investigate the effect of materials on cell viability, a test with AlamarBlue was used. AlamarBlue is a direct indicator of cell health; it detects the level of oxidation during respiration, quantitatively measuring cell viability and cytotoxicity.

During the in vitro cytotoxicity assessment experiment, 2 mL of monocyte suspension was added to each well of a standard 24-well cell culture plate at a concentration of 1 × 10^6^ cells in 1 mL (total 2 × 10^6^ cells) and 10 μL of sample per 1 mL of cell suspension. Incubation was performed at 37 °C and 5% CO_2_ for 6 days. After incubation, supernatant was withdrawn from each well to assess cell viability, leaving 300 μL of cell medium in the well.

AlamarBlue (Sigma-Aldrich, Schnelldorf, Germany) was then added to the wells at a volume ratio of 1:10 and incubated for 3 h at plus 37 °C and 5% CO_2_ in a thermostat incubator. After incubation, 100 μL of cell medium with AlamarBlue in triplicate was added into a 96-well plate. Fluorescence was read using a Tecan Infinite 200Pro automated ELISA reader (Tecan, Grödig, Austria) in the “Top reading” mode. The excitation wavelength was 560 nm and emission wavelength was 590 nm.

### 3.2. Evaluation of Pro-Inflammatory Properties of Samples and Their Influence on Monocyte Activation

The effect of the samples on the activation of human peripheral blood monocytes was evaluated by an enzyme-linked immunosorbent assay (ELISA). The enzyme-linked immunosorbent assay was performed according to the manufacturer’s recommendations (Vector-BEST, Novosibirsk, Russia). For this purpose, supernatant was taken from each well, leaving 300 μL of medium with cells in the well to assess viability according to the method described above. To study immunomodulatory properties, the supernatants were stored at minus 80 °C.

### 3.3. Molecular Modeling of the Interaction of Cucurbit[n]urils with Monocyte Receptors

Three-dimensional protein models for molecular docking were downloaded from the Protein Data Bank (PDB) and imported into the Molegro Virtual Docker (MVD) program, Version 6. PDB structures 6NIH (TLR1) [10], 1ZIW (TLR3) [11], 5NAO (TLR4) [12], 8AR2 (TLR5) [13], and 3W3G (TLR8) [14] were used in this work. Co-crystallized water molecules were removed from the structures during import. The built-in “Detect cavities” tool of the MVD program was used to search for cavities in each of the protein structures with default options. Expressed internal cavities with a volume large enough to include a cucurbit[n]uril (n = 6, 7, 8) molecule were found only for the 6NIH structure containing two protomeric protein chains. The largest of these cavities, enclosed between the protomers, has a volume of 2714 Å3; its geometric center of gravity was taken as the center of the spherical search region for docking positions in structure 6NIH. Structure 3W3G also contains two protomeric protein chains. Due to the lack of obvious internal cavities, two options for localizing the spherical search region were used here: at the common center of gravity of both protomeric subunits of 3W3G, or at the common center of gravity of three co-crystallized 2-acetamido-2-deoxy-β-D-glucopyranose molecules located inside the toroidally coiled protomeric subunit A. The remaining protein structures (1ZIW, 5NAO, 8AR2) are small and include a single protein chain, so spherical search regions were placed at the centers of gravity of these chains. The radii of spherical regions in all cases were assumed to be 15 Å (default value in the MVD program). The location of the search regions relative to the investigated protein structures is shown in Figure 3A–F.

Prototype molecular 3D models of **CB[6]**, **CB[7]**, and **CB[8]** were downloaded from www.chemspider.com as 2D-MOL files, imported into the HyperChem 7 program and, after 2D→3D conversion, optimized by the molecular mechanics method with an MM+ force field. The obtained 3D models of **CB[n]** (n = 6) molecules were further optimized by the composite quantum chemical DFT method B97-3c [37] in the gas phase using the ORCA 5.0.4 program [38]. The nature of the achieved stationary point (energy minimum) was confirmed by the absence of imaginary frequencies of normal oscillations.

The optimized structures **CB[6]**, **CB[7]**, and **CB[8]** were imported into the MVD program as ligands for docking; at the same time, a separate computational project was created for each of the above-mentioned protein structures, including the ligands **CB[6]**, **CB[7]**, and **CB[8]** and a specific protein with a prepared search region. Docking in the MVD program was performed with the MolDock force field [39] with a rigid protein structure. Within each computational design, 200 passes (cycles) of molecular docking were performed using a three-dimensional grid (grid) in the search region. The top five docking positions with the most negative values of the MolDock Docking Score (DS) were saved and then refined using the Nelder–Mead method without using a non-grid. The best docking positions of **CB[6]**, **CB[7]**, and **CB[8]** molecules found in each computational design are shown in Figure 4 and the corresponding DS values along with the amino acid residues interacting with the ligand are presented in Table 2.

### 3.4. Statistical Analysis

Statistical analysis was performed using the STATISTICA 8.0 for Windows program (STATISTICA, RRID: SCR_014213). The Mann–Whitney test and *t*-test for independent groups were used. Data were tested for normality of distribution using the Shapiro–Wilk statistical criterion. Results were considered significant at *** *p* < 0.001, ** *p* < 0.01, and * *p* < 0.05.

## 4. Conclusions

This study investigated the effect of cucurbit[n]uril (n = 6, 7, 8) on the viability of macrophages from different donors, as well as the level of immunotoxicity on human macrophages by analyzing the inflammatory mediators secreted by them by enzyme-linked immunosorbent assay. It was found that the studied samples at the selected concentration (10^−5^ M) do not cause a negative effect on cell viability, which is maintained at a level above 50%. At the same time, the samples do not cause pro-inflammatory activation of monocytic macrophages.

The results of this study indicate that cucurbit[n]urils (n = 6, 7, 8) have the potential to be used as a carrier substance for targeted drug delivery systems without inducing or enhancing inflammatory responses. This property is critical to ensure the safety and efficacy of such agents in the context of medical applications. The lack of stimulation of pro-inflammatory cytokine expression demonstrates the promising biocompatibility of the investigated compounds, which is crucial for their successful clinical use. Moreover, the observation that the concentrations of secreted cytokines generally do not exceed control values may suggest that **CB[6]-**, **CB[7]-**, and **CB[8]-**based drug delivery systems can guide the immune response towards an anti-inflammatory phenotype. The molecular modeling results obtained show that **CB[6]**, **CB[7]**, and **CB[8]** can form associations with different Toll-like receptors, which also confirms good prospects for the development of novel delivery systems based on cucurbit[n]urils.

It should be noted that the methods and approaches used in this study have a number of limitations. To determine cytotoxicity, we used a monocyte–macrophage test system, which is a culture of human peripheral blood monocytes. A natural limitation of such a test system is that monocytes do not have the ability to divide, which makes it impossible to detect possible disturbances in the process of cell division under the influence of research objects. The solution to this problem is the use of dividing cell lines, such as NIH/3T3 and MCF-7, which will be the topic of further research. To assess the pro-inflammatory activity of the research objects, the ELISA method was applied, which, despite its accuracy, has certain sensitivity limits. The production of some cytokines (e.g., TNFα) by monocytes was found to be at the level of the minimum sensitivity threshold of the method set by the manufacturer. A solution may be to assess the expression of the corresponding functional genes responsible for cytokine production. The affinity of **CB[6]**, **CB[7]**, and **CB[8]** with some Toll-like receptors of monocytes (TLR1, TLR3, TLR4, TLR5, and TLR8) was analyzed by the molecular docking method on the rigid conformation of the protein molecule, which is a classical key–lock model. This model allows us to calculate the interaction parameters, but does not take into account the changes in the conformation of the protein molecule under the influence of the research objects. The subject of further research will be the use of the “induced fit” model, which implies a more flexible interaction between the receptor and the molecules of the research objects.

Studies on cucurbit[n]urils (**CB[n]s**) have revealed their significant potential for various applications. One of the most attractive aspects of these compounds lies in their unique ability to act as molecular containers for drugs or other bioactive substances. Exploiting the encapsulating properties of **CB[n]s** offers promising opportunities for the development of innovative drug delivery systems and various biomedical technologies.

## Figures and Tables

**Table 2:** Values of DS evaluation function obtained by docking compounds **CB[6]**, **CB[7]**, and **CB[8]** into different biotargets and amino acid residues forming hydrogen bonds with the ligands.

Biotarget	Ligand	DS ^a^	Amino Acid Residues That Form Hydrogen Bonds ^b^
6NIH	**CB[6]**	−223.0	Lys142B, *Asn163B*, Ser165B, *Tyr275A*
	**CB[7]**	−244.6	Ser165A, *Ser191A*, Lys245B (2)
	**CB[8]**	−266.0	*Ser165A*, *Lys245B*, Ser247B, Tyr275B
1ZIW	**CB[6]**	−217.1	His359 (2)
	**CB[7]**	−237.8	His359 (2)
	**CB[8]**	−250.8	*Asn229*, *Ser254*, Ser256, Ser282, *Tyr283*, Tyr326 (2), Asn328
5NAO	**CB[6]**	−189.4	Ser15
	**CB[7]**	−203.4	Ala26
	**CB[8]**	−211.2	Ser18
8AR2	**CB[6]**	−227.2	Thr659
	**CB[7]**	−230.1	Thr656, Thr659 (2)
	**CB[8]**	−235.2	Thr656, Thr659
3W3G (both protomers)	**CB[6]**	−231.4	*His593B*, Arg619B, Arg650B (3)
	**CB[7]**	−241.8	Arg619B (2), Arg650B (3)
	**CB[8]**	−243.1	Tyr563B, His593B, Asn595B, *Arg650B* (2)
3W3G (one protomer)	**CB[6]**	−242.0	Asp462A, Ser465A, *Arg472A*
	**CB[7]**	−272.5	*Asn262A* (2), Arg375A (2), Arg472A (3)
	**CB[8]**	−277.7	Asn262A, *Asn262A*, Arg375A (2), Arg472A (3)

^a^ Values of the evaluation function are given in conventional units of the MolDock force field. ^b^ Residues forming hydrogen bonds with nitrogen atoms of glycolurilic fragments of cucurbit[n]urils are italicized. If an amino acid residue forms two or more hydrogen bonds with a ligand, their number is indicated in parentheses.

## Data Availability

The data presented in this study are available in this article.
